# Detailed characterization of the *Arthrospira* type species separating commercially grown taxa into the new genus *Limnospira* (Cyanobacteria)

**DOI:** 10.1038/s41598-018-36831-0

**Published:** 2019-01-24

**Authors:** Paulina Nowicka-Krawczyk, Radka Mühlsteinová, Tomáš Hauer

**Affiliations:** 1 University of Łódź, Faculty of Biology and Environmental Protection, Laboratory of Algology and Mycology, Poland, Banacha 12/16 Str, 90-237 Łódź, Poland; 2 University of South Bohemia, Faculty of Science, České Budějovice, Czech Republic, Branišovská 1760, 370 05 České Budějovice, Czech Republic

## Abstract

The genus *Arthrospira* has a long history of being used as a food source in different parts of the world. Its mass cultivation for production of food supplements and additives has contributed to a more detailed study of several species of this genus. In contrast, the type species of the genus (*A. jenneri*), has scarcely been studied. This work adopts a polyphasic approach to thoroughly investigate environmental samples of *A. jenneri*, whose persistent bloom was noticed in an urban reservoir in Poland, Central Europe. The obtained results were compared with strains designated as *A. platensis, A. maxima*, and *A. fusiformis* from several culture collections and other *Arthrospira* records from GenBank. The comparison has shown that *A. jenneri* differs from popular species that are massively utilized commercially with regard to its cell morphology, ultrastructure and ecology, as well as its 16S rRNA gene sequence. Based on our findings, we propose the establishment of a new genus, *Limnospira*, which currently encompasses three species including the massively produced *L*. (*A*.) *fusiformis* and *L*. (*A*.) *maxima* with the type species *Limnospira fusiformis*.

## Introduction

Among the simple trichal cyanobacteria, three genera possess helically-coiled trichomes as a prominent diacritical feature: *Spirulina* Turpin ex Gomont 1892, *Halospirulina* Nübel, Garcia-Pichel et Muyzer 2000, and *Arthrospira* Stizenberger ex Gomont 1892. The latter is a widely-known taxon with long history of use as a food source, for example as dihé in Africa or tecuitlatl in Mexico^1–3^. Presently, the members of this genus are still used as food, but a substantial part of *Arthrospira* production is sold in a form of food supplements or food additives^4,5^; furthermore, under the commercial name of *Spirulina*, it is the most widely-produced microalgae in large-scale production^6^. Latest reports even mention the value of *Arthrospira* in bio-nanotechnology - the coiled shape of its trichomes and their gliding motility with rotation have been used as a biological matrix for creating biohybrid microbots for imaging-guided therapy^7^.

In most industrial applications, *Arthrospira* is used under the common name “Spirulina”, which may be easier to pronounce and remember, and thus appear more suitable from a marketing point of view. However, it is only remotely related to the true *Spirulina*, and despite great efforts, it was not possible to identify any facility producing any species of this genus anywhere in the world. Besides its marketing value, the name is used for historical reasons. In the nomenclatoric starting point for simple trichal cyanobacteria, Gomont^8^ mentions both the genera *Arthrospira* and *Spirulina*. However, *Arthrospira* was not accepted as a separate genus by Geitler^9^ and was hence merged with *Spirulina*. The issue of these two genera was well described by Komárek and Lund^10^, and by Sili and others^6^. Nowadays, a wide array of studies have confirmed that *Arthrospira* and *Spirulina* represent two independent genera, each classified in different orders, and apart from possessing helically-coiled trichomes, the two share a minimal morphological resemblance^11^.

One of the most prominent morphological features of the genus *Arthrospira* is the spiral coiling of multicellular trichomes with easily visible cross-walls^6,12^. The distance between spirals and width of individual coils, both often mentioned in literature as a typical attribute of particular taxa, cannot be considered a permanent morphological feature, since it may change in each trichome. Some authors even describe the ability of this genus to form completely straight and linear trichomes^13,14^. Twenty-three species, either forming mats or living as solitary trichomes, are currently taxonomically accepted within *Arthrospira*^12,15^. Three species are marine, three come from waters with elevated pH and/or electric conductivity, and the rest are freshwater types. The species have been recorded in all continents except Antarctica. In warmer regions, the planktic types often form heavy blooms and are traditionally harvested by local people for direct consumption^2^.

According to the current taxonomic classification, *Arthrospira* is one of the nineteen genera in the Microcoleaceae family within the order Oscillatoriales^11^. Even though several recent studies have dealt with the phylogenetic relationship of *Arthrospira* to other taxa^11,16^, none of these works include molecular data for the type species *A. jenneri* Stizenberger ex Gomont. Unlike more popular species cultivated commercially, *A. jenneri* originates from freshwater ecosystems with low salinity^17^. Strains/organisms determined as *A. maxima* Setchell & Gardner, *A*. *fusiformis* (Voronichin) Komárek & Lund, and *A*. *platensis* Gomont have often been isolated from highly alkaline and saline inland habitats in tropical and subtropical climate zones^18,19^; this knowledge, combined with the lack of information on the type species incorrectly suggests that the preference for high pH and high salinity and/or electrical conductivity is a general feature shared by the entire genus^6^. To better understand not only the ecological requirements of the whole genus *Arthrospira* but also its relationships with other taxa within the order Oscillatoriales, it is necessary to study the type species *A. jenneri* to which the genus name is connected in more detail.

In June 2015, a phytoplankton biodiversity survey of an urban reservoir in Poland (Central Europe) revealed the presence of small mats of *A. jenneri* floating in the water column. Further observations found a thick layer of the organism to be covering the whole bottom of the reservoir. Since then, the presence of *A. jenneri* has been constant: in winter, it creates a visible benthic mat under the ice cover, while in summer, when the air temperature increases to 28 °C, it detaches from the bottom of the reservoir and large lobes of its mat float to the surface.

The present study investigated the relationship of the type species of the genus *Arthrospira*, i.e. *A. jenneri*, to the massively-produced species *A. fusiformis* and *A. maxima*, using traditional and molecular methods. Based upon our findings, we amend the description of the genus *Arthrospira* and delimit the commercially produced species to a new genus, *Limnospira*.

## Results

*Arthrospira jenneri* was first mentioned under the name *Spirillum jenneri* by Hassall in 1845 from Tunbridge, UK. The genus itself was later established by Stizenberger^20^, who compared his own collections from a pond in vicinity of Konstanz, Germany with Hassall’s^17^ description. Gomont^8^, in the literature designated as a starting point for the nomenclature of simple filamentous cyanobacteria, accepts the genus and indicates that this species can be found in various pools and garden lakes in different parts of Europe. Gardner^21^ designated *A. jenneri* as a type species of the whole genus *Arthrospira*, and thus connected the generic name with this organism. None of these authors suggest that *A. jenneri* should inhabit highly saline or alkaline waters. The morphology, ecology and to an extent, the geographical origin of the population of *A. jenneri* collected in Tomaszowska Reservoir, Poland, studied in this paper, correspond well with those given in the original descriptions in previous papers. It can hence be regarded as a reliable candidate for type species of *Arthrospira* in investigating its relationship to other species currently included in this genus.

### Amended description of *A. jenneri* based on the studied population

#### Morphology

**Figure 1:**
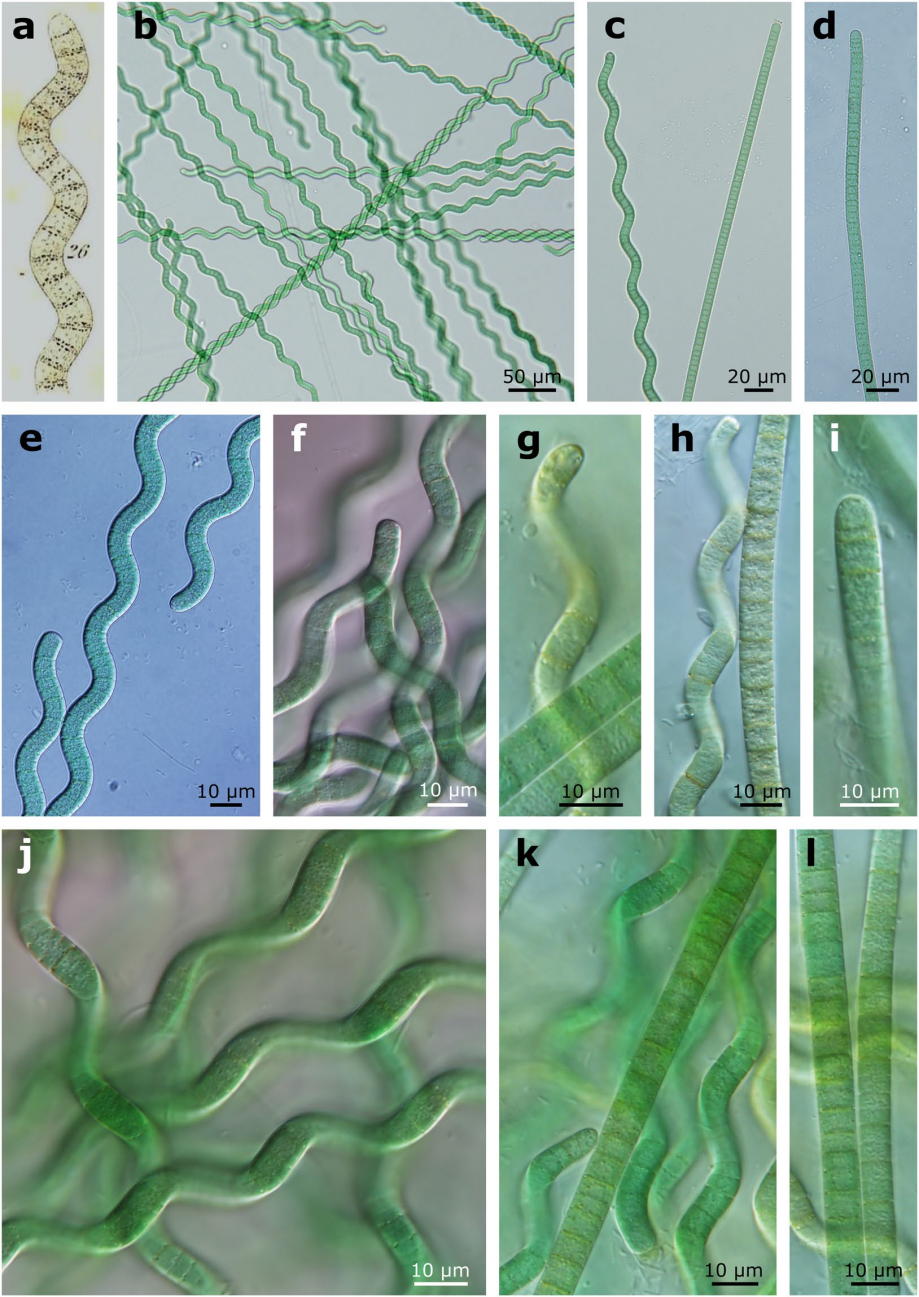
The morphology of *Arthrospira jenneri*, (**a**) original drawing of *A. jenneri* by Gomont^8^, (**b**–**l**) morphology of straight and spiral trichomes of *A. jenneri*’s population from Tomaszowska Reservoir in LM.

**Figure 2:**
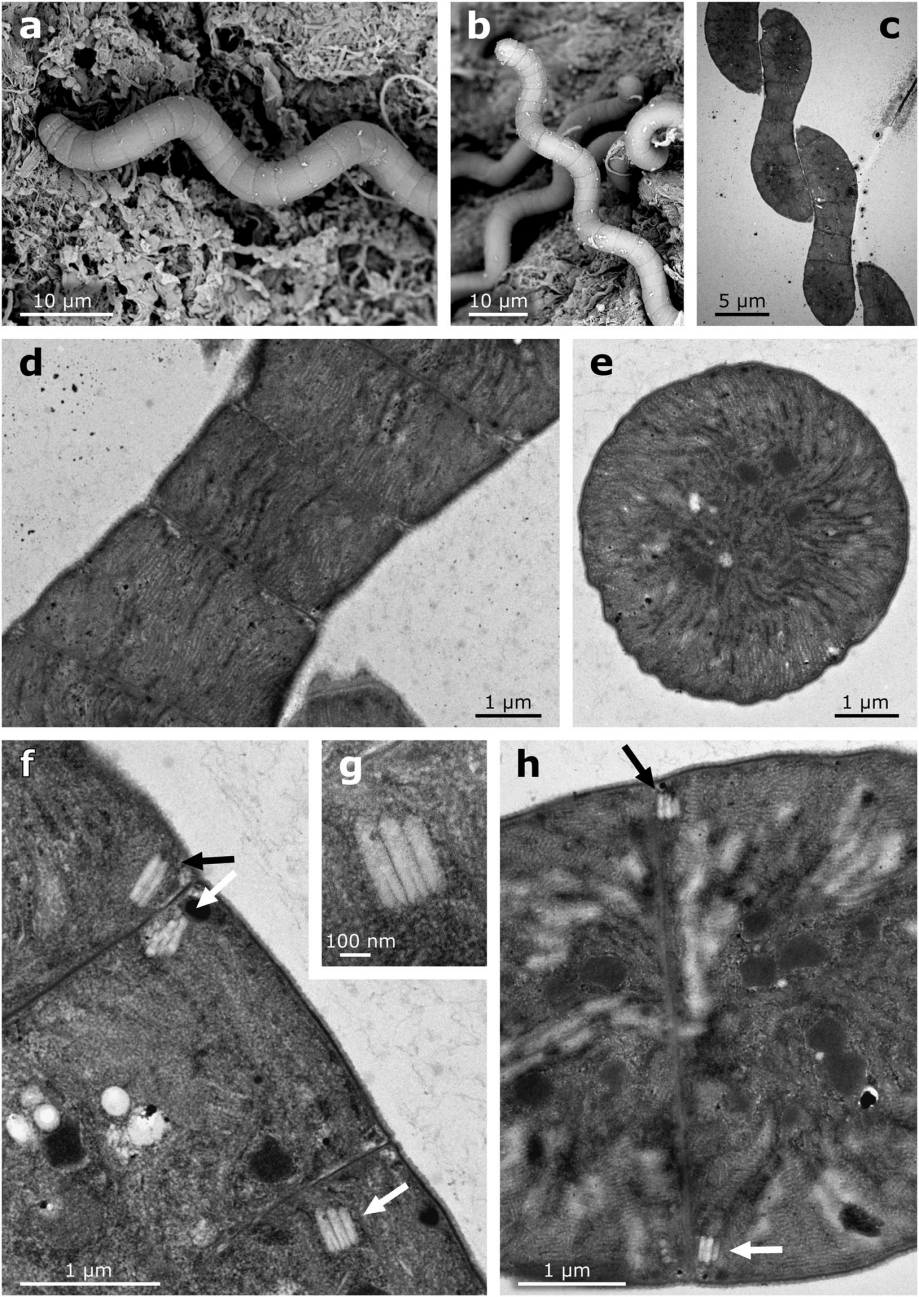
The morphology and ultrastructure of *Arthrospira jenneri* under electron microscope, (**a,b**) morphology of the trichomes in SEM, (**c**–**h**) ultrastructure of the trichomes in TEM, aerotopes indicated by arrows, (**g**) detail of an aerotope.

Trichomes are isopolar, cylindrical, spirally coiled or straight (Fig. 1, Fig. 2a–c). Trichomes of both types (spirally coiled and straight) are motile, unsheathed or with a thin, inconspicuous sheath, not or only slightly constricted at the cross walls and with rounded apical cells lacking calyptra or thickened cell wall (Fig. 1e–i; Fig. 2a,b). Cells of both trichome types are shorter than they are wide; 6–8 µm wide, 2.6–5.6 µm long. Both types of trichomes possess granulation at cross walls (Fig. 1g–i).

#### Ultrastructure

Cells possess radially-arranged thylakoids (Fig. 2e) and facultative gas vesicles forming aerotopes near the cross-walls (Fig. 2f–h).

#### Ecology

Benthic mats in standing waters in temperate zones with optimum growth between pH 6.8 and 7.5, and electrical conductivity between 250 and 445 µS.cm^−1^.

### Phylogenetic analyses

**Figure 3:**
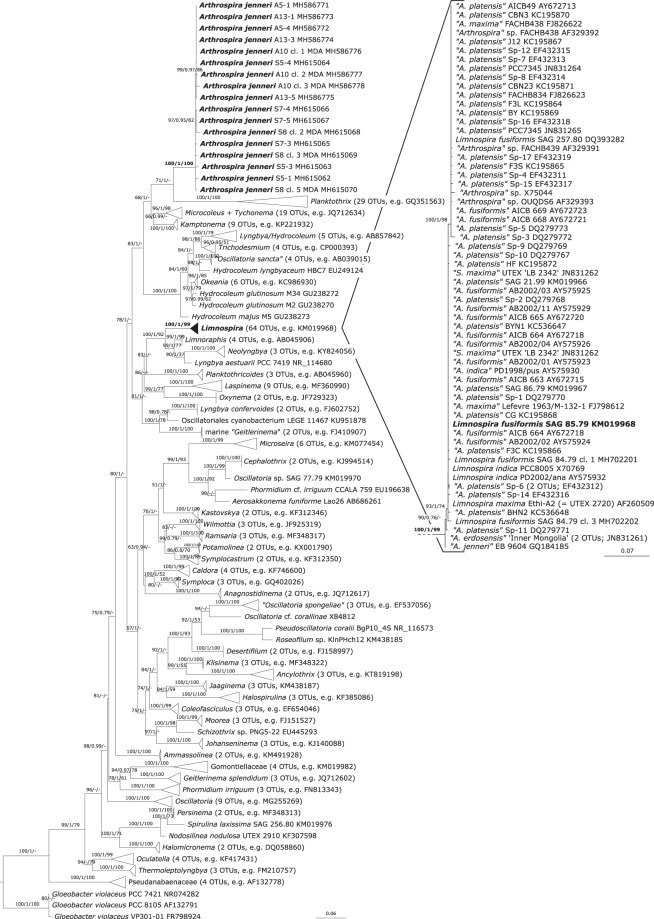
Phylogenetic tree based on 1800 bp fragment of the gene for 16S rRNA, including 291 sequences and showing the position of the genera *Arthrospira* and *Limnospira*. Branch support values (above 50 or 0.75) are shown as Maximum Likelihood, Bayesian posterior probability, and Maximum Parsimony bootstrap values, respectively. Note to Fig. 3: Quotation marks indicate the uncertainty of the real name of the sequenced organism.

Phylogenetic analyses based on the 16S rRNA gene placed all sequences of *A. jenneri* from Tomaszowska Reservoir, Poland, within a well-supported clade well distant from all other sequenced genera (Fig. 3). With regard to the congruence of morphological and ecological data of the sequenced population with the original and starting point descriptions of the type species, this clade represents the genus *Arthrospira*. Maximum Likelihood and Bayesian inference placed the genus *Planktothrix* within the close vicinity of *Arthrospira*. All sequences of widely-consumed taxa in public sequence repositories determined as *A. platensis*, *A. maxima*, or *A. fusiformis* were grouped together with *A. erdosensis*, *A. indica* and* ‘A. jenneri’*, in a well-supported clade, distant from *Arthrospira*. The similarity between the sequences included in these two clades is below 91.21%.

Based on this information, we propose the establishment of a new genus, *Limnospira*, containing, *inter alia*, the mass-produced species of former “*Arthrospira*”. The sequence of *A. jenneri* (GQ184185) located in the *Limnospira* clade originates from organism inhabiting alkaline lakes, and thus cannot represent true *A. jenneri*. All performed analyses suggest that the new genus *Limnospira* is related to two recently-described genera, *Limnoraphis* (freshwater) and *Neolyngbya* (marine), together with the *Lyngbya aestuarii* PCC 7419 strain originating from a salt marsh in the USA (Fig. 3).

***Limnospira*** **gen. nov**.

**Class: Cyanophyceae**

**Order: Oscillatoriales**

**Family: Microcoleaceae**

#### Morphology

**Figure 4:**
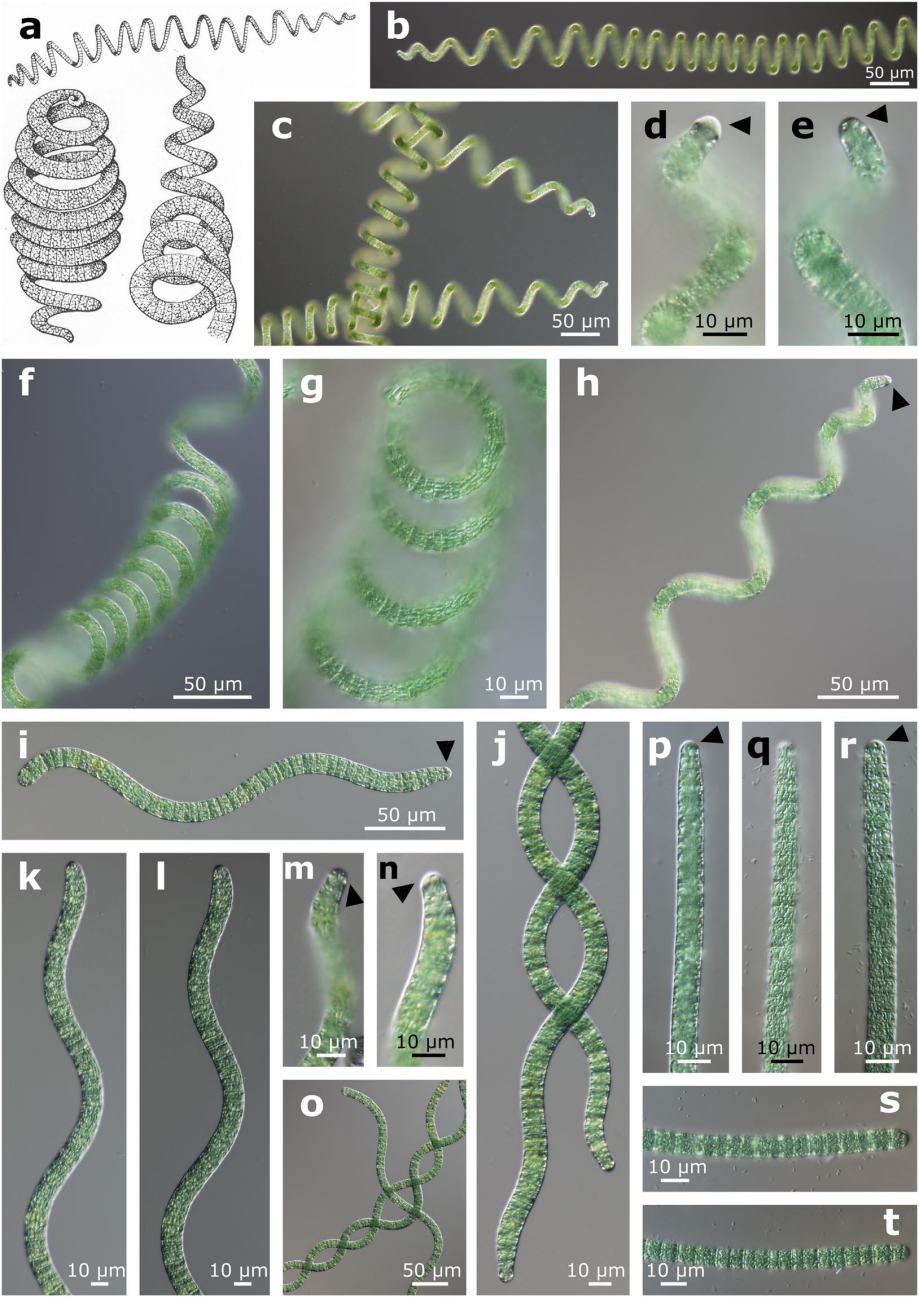
The morphology of *Limnospira*, (**a**) original drawing of *L. fusiformis* (*Spirulina fusiformis*) by Voronichin^22^, (**b**–**h**) morphology of the *L. fusiformis* reference strain SAG 85.79 in LM (**i**–**o**) morphology of the *L. fusiformis* strain SAG 84.79 in LM, (**p**–**t**) morphology of the *L. fusiformis* strain SAG 257.80 in LM; black triangles point out calyptra/thickened cell wall.

**Figure 5:**
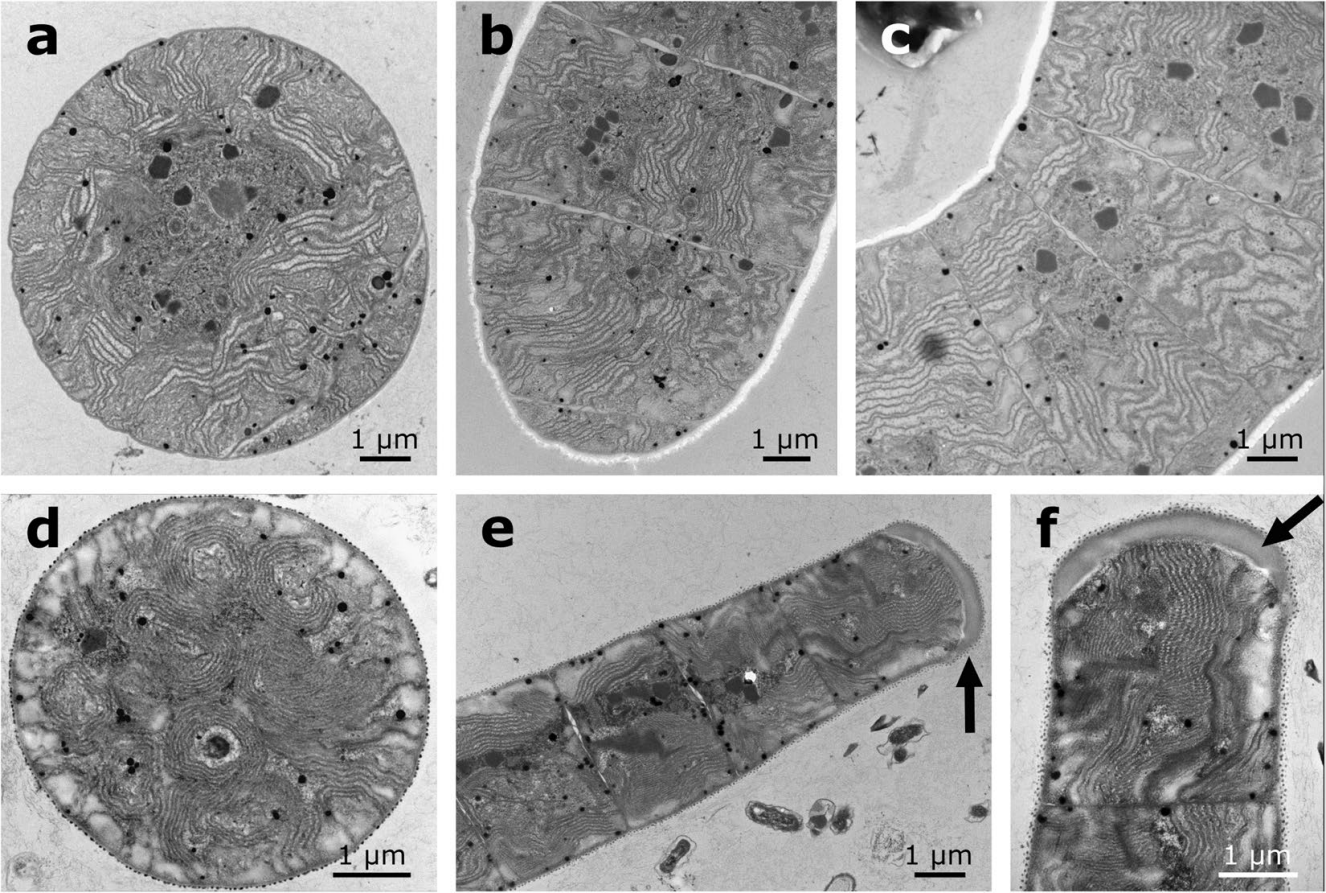
The ultrastructure of *Limnospira* in TEM, (**a**–**c**) the reference strain of *L. fusiformis* SAG 85.79, (**d**–**f**) the strain of *L. fusiformis* SAG 257.80, black arrows point out the calyptra/thickened cell wall at the end of the trichome.

Trichomes isopolar, cylindrical, regularly spirally coiled with a tendency to loosening the coils, unbranched, more or less tapering towards the ends, not or slightly constricted at cross-walls, blue green or dark green. Cells always shorter than wide. Terminal cells rounded or subcapitate with thickened outer cell wall or calyptra (Figs 4d,e,h,i,m,n,p–r and 5e,f).

#### Ultrastructure

Thylakoids have an irregular arrangement (Fig. 5). Aerotopes are facultatively present.

#### Ecology

Planktic in freshwater as well as in water with higher salinity, most of the species known so far prefer habitats with elevated pH and electrical conductivity.

#### Type species

*Limnospira fusiformis* (Voronichin) comb. nov.

#### Etymology

Límni- [Gr., λίμνη], lake, lagoon; -speíra [Gr., σπείρα] - spiral; the generic name refers to the morphology of trichomes and typical habitat, i.e. lakes.

### *Limnospira fusiformis* (Voronichin) comb. nov

#### Basionym

*Spirulina fusiformis* Voronichin 1934, Trudy Soveta Izucheniu Prirodnych Resursov, Ser. Sibirsk. 8: 182^22^ Reference strain: SAG 85.79 = CCALA 026 = UTEX 2340 (Fig. 4b–h, Fig. 5a–c)

### *Limnospira maxima* (Setchell et Gardner) comb. nov

#### Basionym

*Arthrospira maxima* Setchell et Gardner 1917, Univ. Calif. Publ. Bot. 6: 377^23^ Reference strain: UTEX 2720 (sub. “*A. fusiformis”* Ethi-A2).

### *Limnospira indica* (Desikachary et Jeeji Bai) comb. nov

#### Basionym

*Arthrospira indica* Desikachary et Jeeji Bai 1992, ETTA Nat. Symp., Madras: 15^24^ Reference strain: PCC 8005.

## Discussion

Our phylogenetic analyses allowed us to group former *Arthrospira* sequences from main public repositories (64 OTUs) into a single homogenous cluster, closely related to marine *Neolyngbya*, halophilic *Lyngbya aestuarii* PCC 7419 and freshwater cyanobacteria from the genus *Limnoraphis*; while the sequences obtained from both the spiral and straight trichomes of *A. jenneri* (17 OTUs) formed a coherent cluster related to the freshwater genus *Planktothrix* which were distant from the newly-established *Limnospira* (Fig. 3).

Most strains/species forming the genus *Limnospira* are well known for their preference of alkaline habitats (e.g. *L. fusiformis*). This is also one of the characteristics that makes the mass production of the varieties widely used in the food industry easier and less prone to contamination by other organisms^5,6,25,26^. Nevertheless, it seems that the ecological preferences of alkaline waters cannot be generalized for the whole genus. Based on our phylogenetic analyses, sequences representing *A. indica* also belong to the *Limnospira* clade (Fig. 3), and the authors of this taxon and Ballot and others^27^ report that this species comes from a freshwater environment. As it was impossible to track the origin of all of the other sequences grouped in *Limnospira* in the present study, there may be more representatives of this genus that do not require elevated pH for their growth.

The arrangement of thylakoids within the cells and the presence of calyptra/thickened cell wall in apical cells represent more stable and reliable features that can be used to distinguish *Arthrospira* from *Limnospira*. The ultrastructural investigations revealed that *Arthrospira* contains radially-arranged thylakoids (Fig. 2), while *Limnospira* possesses many irregular, whirl-like sections across the central part of the cell (Fig. 5). Whereas the number or density of thylakoids may be modified by environmental factors, the arrangement is very stable in genetic clusters within the 16S rRNA gene^28,29^. Furthermore, the presence of a thickened cell wall or calyptra seems to be present only in the genus *Limnospira* and was not observed in any of the *Arthrospira* trichomes, nor mentioned by the author of the original description of *A. jenneri*.

The current work proposes that only three former *Arthrospira* species be transferred to the newly-established genus *Limnospira*: *L. fusiformis, L. maxima* and *L. indica*. All three possess credible sets of morphological, molecular and ecological data on which their transfer can be based. Unfortunately, many other taxa currently classified as *Arthrospira* lack such necessary data. Despite the fact that many of the sequences forming the *Limnospira* clade are labelled as “*A. platensis*” (Fig. 3), this determination could not be verified by our results. According to the literature, *A. platensis* Gomont represents a benthic organism described from Uruguay with rare, relatively small and inconspicuous broadly rounded calyptras and probably no aerotopes^10,30^. Hence, based only on the presence of calyptra, this species should also be transferred to *Limnospira*. On the other hand, the benthic habitat and apparent absence of aerotopes would suggest a closer proximity to *Arthrospira*, as re-defined here. Regrettably, the available sequences designated as “*A. platensis*” do not seem to originate in the benthic habitats of Uruguay; on the contrary, all seem to represent planktic forms. Therefore, it is not certain whether the available molecular data really represents *A. platensis* and hence, whether the whole species should remain in *Arthrospira* or be transferred elsewhere.

Another example of unclear taxon requiring revision based on more detailed information is the species “*A. erdosensis*”, a type isolated from alkaline ponds in Erdos Plateau, Inner Mongolia, China. “*A. erdosensis*” has not been formally established and insufficient information is available to conclusively place it in a taxon. Hence, it was decided to present most of the names listed in the *Limnospira* clade, as currently named in GenBank/EML ENA/DDBJ, with quotation marks to indicate the uncertainty of their proper name (Fig. 3).

Despite the fact that such high morphological variability occurs in cyanobacteria, and certain morphological features can be used for the identification of taxa, the use of trichome spirality as a diacritical feature on its own may be misleading. As reported by several authors^14^ *Limnospira* is capable of reversible straightening and our results have shown the same for *Arthrospira* sensu stricto. This raises the question whether Hassall’s^17^ note in the very first description of *A. jenneri* “…mixed up with different species of *Oscillatoriæ*.” may apply to straight trichomes of the same species of *Arthrospira*. Non-heterocytous cyanobacteria exhibiting spiral trichomes were divided into three distinct genera outside of the order Synechococcales: the true genus *Arthrospira*, the closely-related *Planktothrix* (Fig. 3), which also contains species characterized by both straight (e.g. *P. agardhii*) and spiral (e.g. *P. spiroides*) trichomes, and the designated genus *Limnospira*. However, none of these genera seem to possess exclusively spiral trichomes, all of them either contain taxa with both coiled and straight trichomes, or the organisms themselves are able to change from one form to another. The only genus where the presence of straight and spiral trichomes has not yet been reported are *Halospirulina* and *Spirulina*, classified to the Synechococcales; however, partial straightening of trichomes has been observed in this case (Komárek pers. comm.). We therefore hypothesize, that some described species of the genus *Oscillatoria* Vaucher ex Gomont may represent the straight trichomes of *Arthrospira* or *Limnospira*, e.g. *Oscillatoria curviceps* Agardh ex Gomont.

The unsuitability of employing trichome spirality as a diacritical feature has also been demonstrated by strain SAG 31.96 maintained in the culture collection under the name *Arthrospira massartii*. Molecular and detailed morphological analyses place this strain within the genus *Planktothrix* Anagnostidis et Komárek and it probably represents species *P. spiroides* originally described from pond in China^31^. Since the strain SAG 31.96 was isolated from a pond with *Microcystis* spp. bloom (http://sagdb.uni-goettingen.de), the ecology does not seem to correspond with that described for *A. massartii* (i.e. clear springs). Hence, based on morphological and ecological data given in previous literature, *A. massartii* may truly belong to the genus *Arthrospira*^12,32^. However, to ultimately confirm whether the organism corresponds closely with the original description, with regard to its morphological and ecological characteristics, it needs to be sequenced.

The long history of the utilization of *Limnospira* under the commercial name “Spirulina” has resulted in many incorrect taxonomic designations. Despite the substantial number of scientific papers highlighting the clear separation of *Arthrospira* from *Spirulina*, *Arthrospira*/*Limnospira platensis* still occurs in many scientific studies under the invalid designation of “Spirulina platensis”^33–35^. Despite its value as a marketing tool, being easier to sell dietary products as “Spirulina” than “Arthrospira”, which calls to mind arthrosis, there is no scientific justification for using this invalid designation. Komárek^29^ argues that such common practice is a serious problem in modern science, and not using the formal nomenclatoric rules undermines the validity of research papers. Unfortunately, in some cases, culture collections also contribute to the confusion surrounding the species level nomenclature by offering strains with incorrect species designations: for example, UTEX LB 2720 is not *L*. (*A*.) *fusiformis* but *L*. (*A*.) *maxima* (according to Li and others^36^) or SAG 85.79 is not *A. platensis* according to its morphology or ecology.

Such nomenclature is also often incorrectly used in the case of commercial products. Biomass producers as well as retail products manufacturers cannot adopt nomenclatoric changes as easily as the scientific community due to several reasons, such as legislation and the need to avoid confusion by the consumer. On the other hand, it would be reasonable to expect them to follow long term trends in taxonomy which may take place over several decades. The polyphasic approach applied recently in cyanobacterial diversity research has resulted in many changes among the genera, families and orders, revealing more authentic phylogenetic relationships. For environmental scientists and specialists in monitoring laboratories who rely almost solely on traditional morphological traits, the molecular approach allows the creation of new taxonomic units which would be impossible to recognize without at least detailed ultrastructural studies. Fortunately, no such analysis is required for the newly-established genus *Limnospira* described herein, since some of its diacritic features are recognizable under the light microscope. Nevertheless, since modern criteria have been accepted within the classification system and have been applied to cyanobacterial taxonomy, it is essential that scientific papers and reports adopt the modern approach and use the names consistent with formal nomenclature.

In conclusion, our detailed investigations of the type species of the genus *Arthrospira* based on a polyphasic approach revealed clear differences between *A. jenneri* and organisms produced under the common name “Spirulina”, hence the need for the establishment of a new genus. We therefore propose the name *Limnospira* for the new genus that encompasses species with high industrial utilization and potential.

## Materials and Methods

### Study material and environmental background

Tomaszowska Reservoir (51°43′48′′N; 19°31′47′′E), where a massive occurrence of *A. jenneri* was noted, is located in the margins of the city of Łódź (Central Poland). It is a flow-through man-made reservoir located on the Olechówka River, with a surface area of 1 ha, average depth of 1.75 m and capacity of 17500 square m^37^. Hydrochemical data is presented in Table S1.

The *A. jenneri* samples were collected from the benthos of the reservoir once every two months for a year, beginning December 2016. Samples for analyses were taken from 25 cm^2^ of the bottom sediment using a plastic frame and small shovel.

Fresh samples of *A. jenneri* were examined by light microscopy (LM), scanning electron microscopy (SEM), and transmission electron microscopy (TEM). In addition, strains of “*A. platensis”* SAG 257.80, SAG 85.79 and “*A. maxima”* SAG 84.79 were analysed in TEM. The LM observations were performed using an Olympus BX51 light microscope equipped with Nomarski DIC optics and an Olympus DP71 digital camera. The surface of trichomes was visualized using a Phenom Pro-X Scanning Electron Microscope. For ultrastructural observations in TEM fresh sample was fixed in 6% glutaraldehyde, left for several hours at room temperature and washed with 0.05 M phosphate buffer (pH 7.2). Then the sample was postfixed with 2% osmium tetroxide in the same buffer and temperature for two hours and repeatedly washed. Trichomes were dehydrated in isopropanol at gradually increasing concentration series and embedded in Spurr’s resign. Ultrathin sections were cut and contrasted with 2.5% uranyl acetate and analysed using a digital JEOL JEM–1010 Transmission Electron Microscope.

### 16S rRNA gene sequencing

Individual trichomes or trichome fragments were isolated from natural material using glass microcapillary as described in Zapomělová and others^38^, and Mareš and others^39^. Every trichome was washed in 6 drops of sterile TE buffer, subsequently placed to sterile 0.2 ml PCR tube and kept frozen (−20 °C) until further use. Three PCR tubes per morphotype were used for the molecular analyses to confirm that the same 16S rRNA is amplified from all of them. Direct PCR was applied to 2 of the PCR tubes using primers 359F^40^ and 23S30R^41^ in 50 μl reaction volume. The PCR mix contained 25 µl of Plain PP Master Mix (Top Bio, Prague, Czech Republic), 1.5 μl of each primer (concentration 5 pmol·μl^−1^), and 21 μl of PCR grade water. The cycling conditions were: 5 min denaturation at 95 °C, continued with 45 cycles of 94 °C for 1 min 30 s, 54 °C for 1 min 30 s, and 72 °C for 2 min, and final elongation at 72 °C for 10 min. The genomic DNA from the third trichome was amplified by the MDA method^42^ using Repli-g Mini Kit (Qiagen, Hilden, Germany) according to the manufacturer’s protocol, followed by PCR using primers 16S27F/f D1^41,43^ and 23S30R^41^ in 20 μl reaction volume. The PCR mix contained 10 µl of Plain PP Master Mix (Top Bio, Prague, Czech Republic), 1.2 μl of each primer (concentration 5 pmol·μl^−1^), and 6.6 μl of PCR grade water. The cycling conditions were: 5 min initial denaturation at 95 °C, continued with 40 cycles of 94 °C for 1 min, 57 °C for 45 s, and 72 °C for 2 min, and a final elongation step at 72 °C for 10 min. After staining by Sybr Green (Lonza, Rockland, ME, USA) the PCR products were cut out of low melting agarose gel (1.5%, 60 V, 1 hour). The bands of interest were cloned into plasmids with pGEM^®^-T Easy vector system (Promega Corp., Madison, WI, USA). Three bacterial colonies containing the PCR product were purified from each plate and then sequenced commercially using following primers: T7 (5′-TAA TAC GAC TCA CTA TAG GG-3′), SP6 (5′-TAT TTA GGT GAC ACT ATA G-3′), and when needed also internal primer CYA781F(a)^40^. The obtained sequences were submitted to the NCBI GenBank database (www.ncbi.nlm.nih.gov): MH586771-78 and MH615062-70. The sequences were assembled in Geneious version 9^44^ (http://www.geneious.com).

### Phylogenetic Analyses

For phylogenetic analysis, the 16S rRNA sequences of *A. jenneri* obtained by us together with other *Arthrospira* records and representatives from simple trichal groups available in GenBank, and the outgroup taxa - *Gloeobacter violaceus* (NR_074282; AF132791; FR798924) were aligned using MAFFT v. 7^45^ (http://mafft.cbrc.jp/alignment/server/). A total of 291 sequences were included in the analysis. Phylogenetic calculations were performed using maximum likelihood analysis (ML) in IQ-TREE web server^46^ (http://iqtree.cibiv.univie.ac.at/), Bayesian inference (BI) in MrBayes 3.2.2^47^ and maximum parsimony analysis (MP) in MEGA 7^48^. The ML tree was constructed applying the GTR + I + Γ model chosen according to Akaike Information Criterion provided by jModelTest 2 software^49^ and 1000 bootstrap replicate searches were performed to evaluate the relative support of branches. The MP involved 1000 replicate searches using the tree bisection-reconnection (TBR) branch-swapping algorithm. A total of 1000 replications were run to evaluate the relative branch support.

## Electronic supplementary material

Table S1

## Data Availability

The sequence data generated during the current study are available in the NCBI GenBank database (www.ncbi.nlm.nih.gov). Other data analysed during this study are included in this article and its Supplementary Information file.
