# Supplementary material for: Detailed characterization of the Arthrospira type species separating commercially grown taxa into the new genus Limnospira (Cyanobacteria)
Source: Sci Rep. 2019 Jan 24;9:694. doi: 10.1038/s41598-018-36831-0 (PMC6345927; doi:10.1038/s41598-018-36831-0)
Supplement: Supplementary file 1 — Table S1 [file 41598_2018_36831_MOESM1_ESM.pdf]

Detailed characterization of the *Arthrospira* type species separating commercially grown taxa into the new genus *Limnospira* (Cyanobacteria)

Paulina Nowicka-Krawczyk, Radka Mühlsteinová, Tomáš Hauer

Table S1. Hydrochemical data of Tomaszowska Reservoir, (a) extremely high value was recorded in spring (April 2017) when road salt was washed out with melting snow, (b) total phosphorus and total nitrogen were measured only once in October 2017.

Parameter	Average	Range
**pH**	7.35	6.8–7.8
**EC [µS.cm^{-1}]**	367	250–445 (640)[(a)]
**P-PO$_4$[mg.l^{-1}]**	0.057	0.045–0.080
**TP(b) [mg.l^{-1}]**	0.26	-
**N-NH$_4$ [mg.l^{-1}]**	0.37	0.22–0.55
**N-NO$_3$ [mg.l^{-1}]**	0.77	0.64–0.92
**TN(b) [mg.l^{-1}]**	2.59	-
